# Neem (*Azadirachta indica* A. Juss) Oil: A Natural Preservative to Control Meat Spoilage

**DOI:** 10.3390/foods4010003

**Published:** 2015-01-09

**Authors:** Paola Del Serrone, Chiara Toniolo, Marcello Nicoletti

**Affiliations:** 1 Agricultural Research Council (CRA), Research Center of Animal Production (CRA-PCM), Via Salaria 31, Monterotondo, RM 00015, Italy; 2 Department of Environmental Biology, University of Rome Sapienza, Piazzale Aldo Moro 5, Rome 00161, Italy; E-Mails: chiara.toniolo@uniroma1.it (C.T.); marcello.nicoletti@uniroma1.it (M.N.)

**Keywords:** neem oil, *Azadirachta indica*, meat spoilage control, antibacterial activity, HPTLC

## Abstract

Plant-derived extracts (PDEs) are a source of biologically-active substances having antimicrobial properties. The aim of this study was to evaluate the potential of neem oil (NO) as a preservative of fresh retail meat. The antibacterial activity of NO against *Carnobacterium maltaromaticum*, *Brochothrix thermosphacta*, *Escherichia coli*, *Pseudomonas fluorescens*, *Lactobacillus curvatus* and *L. sakei* was assessed in a broth model system*.* The bacterial growth inhibition zone (mm) ranged from 18.83 ± 1.18 to 30.00 ± 1.00, as was found by a disc diffusion test with 100 µL NO. The bacterial percent growth reduction ranged from 30.81 ± 2.08 to 99.70 ± 1.53 in the broth microdilution method at different NO concentrations (1:10 to 1:100,000). Viable bacterial cells were detected in experimentally-contaminated meat up to the second day after NO treatment (100 µL NO per 10 g meat), except for *C. maltaromaticum*, which was detected up to the sixth day by PCR and nested PCR with propidium monoazide (PMA™) dye. In comparison to the previously published results, *C. maltaromaticum*, *E. coli*, *L. curvatus* and *L. sakei* appeared more susceptible to NO compared to neem cake extract (NCE) by using a broth model system.

## 1. Introduction 

The post-antibiotic age will be announced when no medicinal drug will be able to successfully fight the multiresistance of microorganisms. Meanwhile, pharmaceutical industries are less interested in developing new antibiotics that can lose their efficacy in a shorter time [1,2]. Therefore, new substances and alternative approaches are urgently requested to face the novel challenges of out-of-control bacteria [3]. Furthermore, our need for novel antibiotics is not limited to medical drugs, but other fields appear very important and crucial for our future. The exploration of plant-derived antimicrobials should be an innovative way to find alternative substances to current antibiotics.

Plants and their agro-industrial waste and by-products constituents are sources of biologically-active substances compared to the current antibiotics. They represent innovative opportunities to control microorganisms in food as alternative to synthetic preservatives [4,5,6]. Spoilage bacteria that negatively influence meat products, causing sour off-flavors, discoloration, gas production, slime production and a decrease in pH, belong to Gram-positive, Gram-negative, anaerobic and facultative genera. These effects have been attributed, among others, to the action of extracellular compounds, such as lipases and proteases, produced by dominant spoilage microorganisms*.* In this paper, some spoilage bacteria were selected with the intention to test the neem oil (NO) antibacterial activity on the basis of the high occurrence and the capacity to act also during the ordinary preservation methods, like low temperature and vacuum.

*Brochothrix thermosphacta* is an economically important psychrotrophic, facultative anaerobic, meat-spoilage microorganism, because it is commonly present in refrigerated meat and meat products packaged in different ways. It produces malodorous metabolic end products, such as acetoin and acetic, isobutyric and isovaleric acids, which make meat unpalatable. This bacterium can display lipolytic activity also at refrigeration temperature [7]. 

The responsible spoilage metabolites of *Carnobacterium* spp. are not structurally well characterized, but branched alcohols and aldehydes play a partial role [8].

*Escherichia coli* presence is considered an indicator of the quality of packed meat. A high presence of *E. coli* (higher than 100 per g) on stored meat could indicate temperature abuse, because it does not grow below 7 °C. *E. coli* presence may also indicate a food safety issue. Its contamination does not cause an odor [9].

Pseudomonas species live in soil, water, on the surface of animal skin, on plants, on many man-made structures and clinical setting [10]. The bacteria in this genus all have strong, durable cell walls. They can utilize diverse compounds, including various hydrocarbons, as carbon and energy sources. This ability is linked to the production of biosurfactants that allow *Pseudomonas* spp. to utilize and degrade fat associated with meat [11].

The lactic acid bacteria (LAB) are implicated in bloating spoilage of vacuum-packed and refrigerated meat products. *Leuconostoc* spp. and *Lactobacillus* spp. genera, involving psychrotrophic lactic acid, cause spoilage of cold-stored, modified-atmosphere-packaged (MAP) and nutrient-rich foods. Food packaging provides an easier distribution and protects food from environmental conditions, such as light, oxygen, moisture, microorganisms, mechanical injury and dust. Active packaging acts by preserving the condition of the packed food and leading to an increase in shelf life and improvement in safety and sensory properties. 

In addition, the application of such packaging methods on the product surface before packaging can create an environment that may delay or even prevent the growth of undesirable organisms. 

Neem (*A. indica*) is a tree of the *Meliaceae* family coming from the Indian subcontinent and actually present worldwide. Neem importance and distribution is increasing all over the world, due to its beneficial properties, as reported by WHO/UNEP1989 [12]. Neem is considered an effective source of environmentally-powerful natural pesticide and considered to be one of the most promising pesticides. It is believed to be one of the trees of the 21st century for its great potential in pest management, environmental protection and medicine [13]. Among the many products obtained from the seeds, NO is the most commercially relevant [14]. The antimicrobial activity of NO quality has already been investigated [15,16].

The efficacy of neem against bacteria affecting meat quality was already investigated using NCE (EtOAc, CH_3_COOCH_2_CH_3_) [17,18]. The metabolomic fingerprint of NCE used in the above mentioned studies was obtained using HPTLC (High Performance Thin-Layer Chromatography) [19,20]. HPTLC, the last evolution of planar chromatography, allows one to detect the majority of the constituents of an extract in an identifying track, named the fingerprint [21]. Plates can be visualized and derivatized in several ways, obtaining multiple pieces of information [22].

The aim of this study was a further assessment of the antibacterial activity of neem using NO instead of NCE and the evaluation of its potential as a meat preservative. 

## 2. Experimental Section 

### 2.1. Bacterial Strains and Growth Conditions 

The spoilage bacteria, namely *Carnobacterium maltaromaticum* (ATCC^®^ 43224™), *Brochothrix themosphacta*, *Escherichia coli*, *Pseudomonas flurescences*, *Lactobacillus curvatus* (ATCC^®^ 25601™) and *Lactobacillus sakei*, were examined in the experiment. *B. thermosphacta*, *E. coli*, *P. fluorescens* and *L. sakei* were previously isolated and characterized [23,24] and then maintained in Microbank™ vials (bioTRADING, Benelux B.V., Mijdrecht, The Netherlands) at −70 °C. All of the lactic acid bacteria (LAB) examined in the experiment did not produce bacteriocins.

B. thermosphacta, *E. coli* and *P. fluorescens* were grew on nutrient agar base (Oxoid Inc., Milan, Italy) at 22 °C for 48 h; trypticasesoy agar (Oxoid Inc.) at 37 °C for 24 h, nutrient agar (Oxoid Inc.) at 26 °C for 48 h and *Pseudomonas* isolation agar (Sigma Aldrich, Milan, Italy) at 35 °C for 24 h according to the manufacturer’s instructions. The LAB were cultivated on Man, Rogosa and Sharpe agar (Oxoid Inc.) and incubated at 35 °C for 48 h.

Standard bacterial inocula were made with suitable broth culture for each bacterium diluted to match an equivalent 0.5 McFarland turbidity.

### 2.2. Neem Oil

A commercial NO produced by Neem Italia (Manerba (BS), Italy) was used as the test starting material (0.35% azadirachtin A). The total composition of the oil was checked by high performance thin layer chromatography [21].

NO was diluted in Tween 80^®^ (1:1 V/V; VWR, PBI International, Milan, Italy) under agitation and sterilized by filtration through a 0.22 μm Millipore express filter (Millex-GP, Bedford, OH, USA).

### 2.3. HPTLC Assay 

#### 2.3.1. HPTLC System and Materials

The HPTLC system (CAMAG, Muttenz, Switzerland) consisted of: (1) the Linomat 5 sample applicator using 100 µL syringes and connected to a nitrogen tank; (2) ADC2-chamber containing twin trough chamber 20 × 10 cm; (3) Immersion Device III; (4) TLC Plate Heater III; (5) TLC visualizer; and (6) TLC scanner 3 linked to win CATS software.

Solvents for extraction and HPLC-grade solvents were purchased from Sigma-Aldrich and Carlo Erba (Milan, Italy). Glass plates 20 cm × 10 cm with glass-backed layer silica gel 60 (2 μm thickness) were obtained from Merck (Darmstadt, Germany). Before use, plates were prewashed with methanol and dried for 3 min at 100 °C. Standards used in the HPTLC analysis were isolated from neem cake (*i.e.*, salannin, azadirachtin A, unsaturated and saturated lipids) in the Laboratory for Quality Control of the Department of Environmental Biology, University of Rome Sapienza, Italy. 

#### 2.3.2. Sample Application

Filtered solutions were applied with nitrogen flow. The operating conditions were: syringe delivery speed, 10 s μL^−1^ (100 nL s^−1^); injection volume, 2 μL; band width, 6 mm; distance from bottom, 15 mm.

#### 2.3.3. Development

The HPTLC plates were developed in the automatic and reproducibly developing chamber, ADC 2, saturated with the same mobile phase for 20 min at room temperature. The developing solvents (*i.e.*, type of solvents and ratios) were carefully optimized before the analyses. The length of the chromatogram run was 80 mm from the point of application. The developed layers were allowed to dry in air for 5 min, derivatized with a selected solution, including anhysaldehyde (1.5 mL *p*-anisaldehyde, 2.5 mL H_2_SO_4_, 1 mL AcOH in 37 mL EtOH), dried in the open air and then dipped into Macrogol reagent (1 g polyethylene glycol 400 in 20 mL of dichloromethane). Finally, the plates were warmed for 5 min at 120 °C before inspection. All treated plates were inspected by a CAMAG TLC visualizer under a UV light at 254 or 366 nm or under reflectance and transmission white light (WRT), before and after derivatization.

### 2.4. Assessment of Antimicrobial Activity

The antimicrobial activity of NO was assessed by a broth model, as previously reported [18]. It consists of three steps: growth inhibition on solid medium using the disk diffusion method (Whatman filter paper No. 1 discs of 6 mm in diameter impregnated with 100 μL of NO solution; three discs per plate; three plates per each bacterium; one positive control: ciprofloxacin antibiotic (ciprofloxacin hydrochloride monohydrate, 1 mg/mL, Bayer, Milano, Italy), sterile distilled water and Tween^®^ 80 (VWR International PBI Srl, Milano, Italy, 1 mg/mL) as negative controls; the experiment was performed twice; growth reduction in liquid medium using the broth microdilution method (using conventional sterile polystyrene microplates of 96 wells; each well was filled with 100 μL of sterile suitable liquid media for each microorganism considered, 50 μL of inoculums and the amounts of extract at lower concentrations 1:10 to 1:10,000); minced vacuum-packed meat (10 g) experimentally inoculated with each bacterium (*ca*. 10^4^ CFU/mL) and treatment with NO (10 µL), sterile distilled water and ciprofloxacin (100 µg) (CFX) as controls. 

Viable bacterial cells were detected and identified directly in experimentally-treated samples at two-day intervals up to the 12th day of refrigerated storage at 10 °C to simulate an abusive refrigeration; and detection by direct and nested PCR with PMA dye (PMA™ Biotium Inc., Hayward, CA, USA, www.biotium.com). 

The results were recorded as the means ± SD of the duplicate experiment considering three repetitions for each experiment. Differences between the means of data were compared by least significant difference (LSD) calculated using the Statistical Analysis System (SAS 2000). 

### 2.5. Molecular Biology Assay

The primer pairs that amplify species-specific genomic sequences: *Carnobacterium* spp. Cb1 (5′-CCG TCA GGG GAT GAG CAG TTA C-3′)/Cb2r (5′-ACA TTC GGA AAC GGA TGC TAA T-3′) [25]; *B. thermosphacta* pA (5′-AGA GTT GAT CCT GCC TCA G-3′)/pE (5′-CCG TCA ATT CCT TTG AGT TT-3′); *E. coli* Ec1, (5′-CCG ATA CGC TGC CAA TCA GT-3′)/Ec2 (5′-ACG CAG ACC GTA AGG GCC AGA T-3′) [26]; *P. fluorescens* 16SPSEfluF (5′-TGC ATT CAA AAC TGA CTG-3′)/16SPSER (5′-AAT CAC ACC GTG GTA ACC G-3′) [27]; *L. curvatus* Y1 (5′-TGG CTC AGA ACG AAC GCT GGC CCG-3′)/Y2 (5′-CCC ACT GCT GCC TCC CGT AGG AGT-3′) and 16S (5′-GCT GGA TCA CCT CCT TTC-3′)/Lc (5′-TTG GTA CTA TTT AAT TCT TAG-3′)[27]; and *L. sakei* Y1 (5′-TGG CTC AGA ACG AAC GCT GGC CCG-3′)/Y2 (5′-CCC ACT GCT GCC TCC CGT AGG AGT-3′) and 16S (5′-GCT GGA TCA CCT TTC-3′)/Ls (5′-ATG AAA CTA TTA AAT TGG TAC-3′) [28] were used in direct PCR and nested PCR to detect viable cells in experimentally-inoculated minced meat and treated with NO, sterile distilled water and ciprofloxacin as controls. The reaction mixtures and conditions of amplification were as reported in the literature. 

The selective dye, a photo-reactive dye with high affinity for DNA that intercalates into DNA and forms a covalent linkage upon exposure to intense visible light, was used for the detection of live cells [29].

The PCR products (7 µL) were loaded on 1.5% to 3% agarose gels (Life Technologies, Monza MB, Italy), with electrophoresis at 5 V cm^−1^ and visualized under UV. After staining the gel with ethidium bromide (Sigma Aldrich, Milan, Italy), a 100 bp ladder (Gibco BRL, Monza MB, Italy) was used as a size marker.

## 3. Results and Discussion

The obtained results show that NO has a broad spectrum of antibacterial activity. Antibacterial activity was evaluated based on the diameters of the clear inhibition zone surrounding the paper disks soaked with 100 µL of NO. As presented in Table 1, the average of the growth inhibition zone (IZ mm) ranged from 18.83 ± 1.18 to 30.00 ± 1.00. Significant differences concerning the growth IZ (mm) between NO and CFX were found among the examined bacteria, with the exception of *P. fluorescens*. *E. coli* resulted the least susceptible among the tested bacteria.

**Table 1:** Antibacterial activity of NO against spoilage bacteria detected by the disc diffusion method as the inhibition zone of growth (mm).

Bacteria	Growth Inhibition Zone (mm) *			
	Treatment			
	NO (100 µL)	TWN (100 µL)	CFX (100 µL)	WTR (100 µL)
*Carnobacterium maltaromaticum*	24.33 ± 0.58 b	-	29.00 ± 1.00 a	-
*Brochothrix thermosphacta*	26.53 ± 1.15 b	-	29.27 ± 1.00 a	-
*Escherichia coli*	18.83 ± 1.18 b	-	27.71 ± 1.00 a	-
*Pseudomonas fluorescens*	30.00 ± 1.00 a	-	30.33 ± 1.73 a	-
*Lactobacillus curvatus*	26.33 ± 0.58 b	-	28.41 ± 1.00 a	-
*Lactobacillus sakei*	27.50 ± 0.50 b	-	29.33 ± 2.08 a	-

* Diameter of inhibition zones, including the diameter of the disc (6 mm). NO, neem oil; TWN, Tween^®^ 80; CFX, ciprofloxacin (1 mg/mL); WTR, sterile distilled water. Three disks papers per plate and three plates for each bacterium were considered. The experiment was repeated twice. -, absence of inhibition zone. Values expressed are as the mean ± standard deviation of two experiments. Mean values with a different letter in the row are significantly different (*p* ≤ 0.05).

The highest bacterial growth reductions (GR%) were observed with 100 µL and 10 µL NO concentrations. They ranged from 84.51 ± 1.15 to 99.70 ± 1.53 and from 86.61 ± 1.00 to 91.73 ± 2.08, respectively. There was no significant differences in the percent of the bacterial growth reduction revealed at 100 µL and 10 µL NO concentrations. In Table 2, there are no significant differences for *L. curvatus* (Table 2).

**Table 2:** Bacterial growth reduction (GR%) at 24 h in liquid medium with differences in the percentage of bacterial growth reduction at different concentrations of NO using the control treatment as reference (without NO).

Bacteria	Growth reduction (GR%)			
	Treatment			
	NSO (100 µL)	NSO (10 µL)	NSO (1 µL)	NSO (0.1 µL)
*Carnobacterium maltaromaticum*	89.65± 1.53 c	88.61 ± 1.00 c	67.67± 1.33 b	30.81± 2.08 a
*Brochothrix thermosphacta*	84.51 ± 1.15 c	89.70± 1.00 bc	67.58± 1.33 ab	34.86± 1.00 a
*Escherichia coli*	91.51 ± 1.15 c	89.70± 1.00 bc	67.58± 1.33 ab	64.86± 1.00 a
*Pseudomonas fluorescens*	88.90± 1.00 c	88.79± 1.00 abc	69.60± 0.00 ab	66.68± 1.20 a
*Lactobacillus curvatus*	99.70 ± 1.53 c	91.73 ± 2.08 bc	68.69 ± 2.00 b	62.83 ± 1.73 a
*Lactobacillus sakei*	89.71 ± 1.00 c	89.61± 0.58 abc	69.57 ± 0.00 ab	67.58 ± 0.89 a

NO, neem oil. Values expressed as the mean ± standard deviation of two experiments (three repetitions for each experiment). Mean values with different letters in the column are significantly different (*p* ≤ 0.05).

Bacterial DNA amplification products of the expected sizes were detected according to Del Serrone and Nicoletti [18] directly in experimentally-inoculated minced vacuum-packed meat up to the second day after NO treatment with the exception of *C. maltaromaticum*, which was detected up to six days after NO treatment. Bacteria were always detected in the control samples (water) at the 2nd, 4th, 6th, 8th, 10th and 12th storage days, but never in samples treated with ciprofloxacin collected on the same storage days (Table 3).

**Table 3:** Detection and identification by PCR and nested PCR of the tested bacterial strain viable cells in vacuum-packed minced beef meat stored at 10 °C at 2, 4, 6, 8, 10 and 12 days after treatment with and without ciprofloxacin.

Bacteria	Detection of viable cells after treatments																	
	2 days			4 days			6 days			8 days			10 days			12 days		
	NO	W	CFX	NO	W	CFX	NO	W	CFX	NO	W	CFX	NO	W	CFX	NO	W	CFX
*Carnobacterium maltaromaticum*	+	+	−	+	+	−	+	+	−	−	+	−	−	+	−	−	+	−
*Brochothrix thermosphacta*	+	+	−	−	+	−	−	+	−	−	+	−	−	+	−	−	+	−
*Escherichia coli*	+	+	−	−	+	−	−	+	−	−	+	−	−	+	−	−	+	−
*Pseudomonas fluorescens*	+	+	−	−	+	−	−	+	−	−	+	−	−	+	−	−	+	−
*Lactobacillus curvatus*	+	+	−	−	+	−	−	+	−	−	+	−	−	+	−	−	+	−
*Lactobacillus sakei*	+	+	−	−	+	−	−	+	−	−	+	−	−	+	−	−	+	−

NO, neem oil; W, sterile distilled Water; CFX, ciprofloxacin. +, detected; −, not detected.

In comparison to the previously published results, *C. maltaromaticum*, *E. coli*, *L. curvatus* and *L. sakei* resulted more susceptible to NO rather than to NCE using the broth model meat system method.

**Figure 1:**
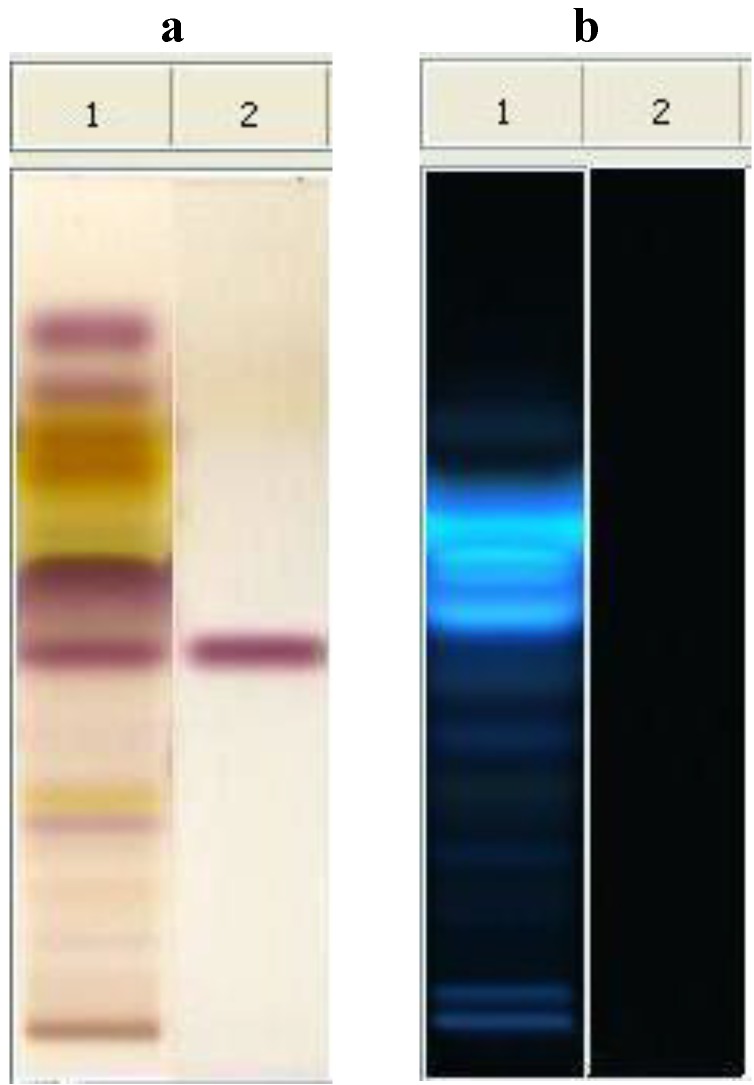
HPTLC analysis of neem oil EtOAc extract. Mobile phase: toluene: AcOEt 7:3 (v/v). Visualization: plate a (left): white light upper and lower; plate b (right): UV lamp at 366 nm. Derivatization: anhysaldehyde. Track 1, neem oil; Track 2, salannin.

The metabolomic fingerprint of NO shows a characteristic sequence of metabolites according to the polarity of constituents [17,18]. The identification of the raw material was assured by the presence of salannin (Rf = 0.42), which is a typical marker of neem. In comparison with the spot of azadirachtin (Rf = 0.23), salannin appears as the main limonoid spot. Spots concerning lipids are present at Rf values at *ca*. 0.80, due to unsaturated fatty acids and fatty alcohols, and at Rf *ca*. 0.50, due to saturated and unsaturated triglycerides [20]. The most interesting feature of the plate concerns the presence of compounds with high fluorescent reaction for Rf 0.55–0.66, which are perfectly visible at 366 nm after derivatization with anhysaldehyde (Figure 1). These spots can be attributed to compounds with high conjugated unsaturation in polycyclic aromatic structures, very different from those of the nortriterpenes limonoids, so far considered as responsible for the activity. Therefore, more studies are necessary to decide the importance of the antibacterial activity of these substances in the phytocomplex [19]. 

## 4. Conclusions

The exploration of PDEs as alternative antimicrobials and preservatives in the food industry is continuously increasing. They represent a lower perceived risk to the consumer. They satisfy increased consumer demand for minimally-processed and synthetic preservative-free food products. The exploration of PDEs as antimicrobials is an innovative way to find new alternative substances as food preservatives for antimicrobial packaging, in order to represent a lower perceived risk to the consumer and to satisfy increased consumer demand for minimally-processed and synthetic preservative-free food products. NO is eco-friendly and target tailored, besides being effective. The broth meat model system employed here allowed for screening the potential preservative quality of plant extracts directly on meat [30,31,32].

The fingerprint of plant-derived extracts or phytocomplex is useful to standardize products and to establish the scientific evidence of their biological activity. World Health Organization recognized the HPTLC fingerprint as a method to identify a plant or its preparations by a characteristic chromatographic profile, where it is not possible to identify a single discriminating active principle.

Currently, the different types of commercial neem cake on the market are roughly identified as oiled and deoiled cake, but several other differences can be detected [33]. The quality of the neem oil, as that of the cake, depends on the quality of the seeds, as well as the type of extraction processes used, which strongly influence the chemical composition of the product. NO has a characteristic odor, but at the level used in the experiment, this was not perceived. However, it is necessary to implement organoleptic tests to demonstrate that it has no negative effect on treated meat.

The antimicrobial activity may be determined by three main methods, disk diffusion, agar dilution and broth macrodilution or microdilution, according to antibiotic susceptibly tests as standardized by international committees to assay antibiotic microbial susceptibility. 

Besides the applied methods, the results of the antimicrobial activity tests can be affected by many other factors, such as the microorganisms tested and the degree of solubility of each test plant-derived extract. Furthermore, the main limitations of the use of antimicrobials to activate films for meat packaging include the inactivation of compounds in contact with the meat surface and their dispersion from the surface into the meat mass. The broth meat model system has previously been developed as a methodology to test the antibacterial efficacy of plant-derived extracts directly on meat, as well as their potential as preservatives for antimicrobial packaging of fresh retail meat. 

The obtained results show that the antimicrobial activity of NO against spoilage bacteria is very promising and is higher compared to the reported antimicrobial activity of NCE. Neem leaf oil is reported to inhibit *Penicillium verrucosum* and *P. brevicompactum* growth, sporulation and mycotoxin (ochratoxin A) production in dry-cured meat products [34], too. In addition, neem extracts are also reported to possess intense antimicrobial properties against bacteria of human interest [35].

Further investigation should be developed on using food packaging, to improve shelf life and to assure the safety of meat products.
